# Phenology-mediated effects of phenotype on the probability of social polygyny and its fitness consequences in a migratory passerine

**DOI:** 10.1186/s12862-021-01786-w

**Published:** 2021-04-13

**Authors:** David Canal, Lotte Schlicht, Simone Santoro, Carlos Camacho, Jesús Martínez-Padilla, Jaime Potti

**Affiliations:** 1 Institute of Ecology and Botany, Centre for Ecological Research, Alkotmány u. 2-4, 2163 Vácrátót, Hungary; 2 Department of Behavioural Ecology and Evolutionary Genetics, Max Planck Institute for Ornithology, Eberhard- Gwinner-Str. 7, 82319 Seewiesen, Germany; 3 Department of Integrated Sciences, Faculty of Experimental Sciences, University of Huelva, Avda de las Fuerzas Armadas s/n, 21007 Huelva, Spain; 4 Department of Biological Conservation and Ecosystem Restoration, Pyrenean Institute of Ecology (IPE-CSIC), Nuestra Señora de la Victoria, 16. 22700, Jaca, Spain; 5 Department of Evolutionary Ecology, Estación Biológica de Doñana (CSIC), Américo Vespucio 26, 41092 Seville, Spain

**Keywords:** Social polygamy, Polygyny, Phenotype, Fitness, Neighbourhood, Polygyny threshold model, Sexy son hypothesis

## Abstract

Why females engage in social polygyny remains an unresolved question in species where the resources provided by males maximize female fitness. In these systems, the ability of males to access several females, as well as the willingness of females to mate with an already mated male, and the benefits of this choice, may be constrained by the socio-ecological factors experienced at the local scale. Here, we used a 19-year dataset from an individual-monitored population of pied flycatchers (*Ficedula hypoleuca*) to establish local networks of breeding pairs. Then, we examined whether the probability of becoming socially polygynous and of mating with an already mated male (thus becoming a secondary female) is influenced by morphological and sexual traits as proxies of individual quality relative to the neighbours. We also evaluated whether social polygyny is adaptive for females by examining the effect of females’ mating status (polygamously-mated vs monogamously-mated) on direct (number of recruits in a given season) and indirect (lifetime number of fledglings produced by these recruits) fitness benefits. The phenotypic quality of individuals, by influencing their breeding asynchrony relative to their neighbours, mediated the probability of being involved in a polygynous event. Individuals in middle-age (2–3 years), with large wings and, in the case of males, with conspicuous sexual traits, started to breed earlier than their neighbours. By breeding locally early, males increased their chances of becoming polygynous, while females reduced their chances of mating with an already mated male. Our results suggest that secondary females may compensate the fitness costs, if any, of sharing a mate, since their number of descendants did not differ from monogamous females. We emphasize the need of accounting for local breeding settings (ecological, social, spatial, and temporal) and the phenotypic composition of neighbours to understand individual mating decisions.

**Supplementary Information:**

The online version contains supplementary material available at 10.1186/s12862-021-01786-w.

## Introduction

Sexual conflict and sexual selection, strengthened by direct and indirect costs and benefits, drive mate choice and the evolution of mating systems [1–4]. Social polygyny, in which one male socially bonds with more than one female over the breeding season, is a common mating system in vertebrates (e.g. [1, 2, 5, 6]). Attracting several females is assumed to be beneficial for males in terms of lifetime reproductive success [7, 8]. However, in species where males provide breeding resources to the mate and offspring, the benefits to females that choose an already mated male are less obvious [2, 9–12]. The evolutionary mechanisms that cause and maintain social polygyny have thus prompted considerable theoretical and empirical research (e.g. [3, 13–17]), either based on adaptive (e.g. sexy son hypothesis; [14]) or non-adaptive scenarios (e.g. deception hypothesis; [18]). Unsurprisingly, why females mate with already-mated males remains a matter of debate (e.g. [4, 13, 15, 19]).

In birds, males in resource-defence polygynous systems have to compete for the acquisition and defence of territories, and for attracting successive females [2, 7]. Males displaying enhanced versions of their sexual traits are expected to be positively selected because of their higher quality and are, therefore, more likely to become polygynous than less attractive males [20–22]. However, studies examining the association between male phenotype and success in polygyny have yielded mixed results [20–22]. Most of these studies have implicitly assumed (e.g. by adopting a population-scale approach) that all individuals experience similar mating opportunities and/or that they can potentially interact and mate with any other female in the population, which are unrealistic assumptions. Mating opportunities vary temporally and spatially according to socio-ecological factors and therefore differ among individuals [1, 4, 23]. Indeed, growing evidence indicates that local context can strongly influence the mating strategies of individuals by determining their relative attractiveness, their competitive ability and the actual number of accessible mates [24–29]. Thus, accounting for the specific breeding settings experienced by individuals is essential to avoid misleading conclusions about the phenotypic, ecological and/or social factors driving mate choice, including those involved in social polygyny. However, exploring the local social context at the breeding grounds poses major logistic and analytical challenges for field-based studies. First, it is necessary to measure both the phenotype and local context of all individuals that could interact (temporally and spatially) with the focal individual(s). Second, it is not straightforward to merge these aspects into a common analytical framework [26–28]. Consequently, very few studies have comprehensively investigated the influence of breeding settings (ecological, social, spatial, and temporal) on mate choice (see, e.g. [26–28]), and particularly in the context of polygyny [30].

While fine-tuned analyses of local breeding conditions are needed to understand variation of polygyny, a full comprehension of its persistence in a population requires quantification of its direct and indirect fitness consequences. From an adaptive perspective, social polygyny should be favoured when the costs of sharing breeding resources (e.g. parental care) for a polygamously-mated female are offset in terms of fitness benefits directly or indirectly [3, 14, 17, 31]. Thus, a rigorous evaluation of the costs and benefits accrued by females through social polygyny requires accurate measurements of the number of offspring (direct) and also the number of grand-offspring (indirect fitness benefits). The latter, however, has rarely been examined [12, 32] because gathering sufficient data on the lifetime reproductive success of females’ offspring to allow for comparisons between mating status (e.g. monogamous *vs.* secondary females) is extremely challenging in wild populations.

Here, we used the pied flycatcher (*Ficedula hypoleuca*) as a model system to investigate whether an individual’s quality is related to the probability of mating polygynously in both males and females, and to determine the fitness consequences for females of mating monogamously or polygamously. We first characterized local networks of breeding pairs. By doing so, it is possible to simultaneously consider the male, female, and pair characteristics, as well as their local breeding contexts (e.g. number of neighbours or the phenotypic composition of the neighbourhood), so that their relative influence on the chances of being involved in socially polygynous matings can be ‘individually’ assessed [26]. We then investigated whether social polygyny is adaptive for females by testing whether direct or indirect fitness measurements of polygamously-mated females equal or exceed those of monogamously-mated females. Based on previous studies on this and other socially polygamous species (see “Study system”), we formulate the following predictions. First, since several traits indicative of quality are favoured in intra- and inter-sexual contexts (e.g. [33–35]), we expect polygynous males, capable of monopolizing several nests and females, to be of higher phenotypic quality than monogamous neighbouring individuals (see Table 1). Second, assuming that (i) females benefit from breeding as early as their condition permits ([36, 37], but see [38]), (ii) primary females of polygynous males settle earlier than secondary females, and (iii) territory quality, competitive ability and productivity often decline throughout the season [16, 23], we also expect differences in individual quality among females of polygynous males (see Table 1; [23, 39]). Third, we expect a lower fitness of secondary females relative to monogamous females, even when the chances of becoming socially polygamous are related to the males’ individual quality, because secondary females are expected to be low-quality individuals (see above) and social polygyny may primarily results from sexual conflict [18]. Alternatively, secondary females may not suffer the potential costs of sharing breeding resources or may be able to offset them, either through direct or indirect (via offspring) fitness.

**Table 1:** Expected effect (positive "+" or negative "−") of the analysed traits on the probability of becoming polygynous and on the probability of becoming secondary female (and thus mating with an already mated male). See main text for further details

Trait	Male	Female
Tarsus length	+	−
Wing length	+	−
Age	+At middle age classes −At yearling and old ages	−At middle age classes +At yearling and old ages
Male’s black plumage (dorsal)	+	*NA*
Male’s forehead patch size	+	*NA*
Number of neighbours	−	+
Local breeding date	+	−

## Material and methods

### Study system

The pied flycatcher is a sexually dimorphic (in plumage and slightly in size; [37, 40]), single-brooded and long-distance migrant passerine that establishes a small territory around a nest hole for breeding [37]. Males arrive at the breeding areas before females, search for a suitable nesting site, defend its possession and try to attract a female, which usually visits several males before settling [36, 41]. The species is predominantly monogamous, but some males (< 25%) occupy more than one nest cavity in different territories, attract additional females and become socially polygamous [30, 38, 42]. Mating with polygynous males can be costly for both primary and secondary females, mainly because of reduced parental care compared to monogamous mated females [32, 37, 42–44]. Further, several male traits, indicative of their quality (e.g. forehead patch size or plumage colour), are sexually selected in the species [34, 35, 45]. These aspects make the pied flycatcher a suitable model system to empirically investigate intrinsic and extrinsic determinants of polygamous matings and their fitness consequences for polygamously-mated females.

### Study population and general procedures

Between 1995 and 2016, we studied a population of pied flycatchers breeding in nest-boxes in central Spain (ca. 41°4’42’’ N, 3°25’55’’ W, 1200–1300 m asl). We used data from 19 breeding seasons, as field effort was limited in 1996, 2002 and 2003 and these years were excluded from analyses.

Field protocols have been described in detail elsewhere [45, 46]. Briefly, georeferenced nest-boxes were regularly checked during the breeding season (April–July) to determine egg-laying date (date of the first laid egg), clutch size, hatching date, and number of offspring. Breeding adults were captured at the nest while incubating (only females) or feeding nestlings (both sexes) at 8–10 days post-hatching. Nests where the male could not be captured during the first capture session were observed during periods of 30–60 min on successive days until fledging (ca. 15-days old), unless a male was observed repeatedly assisting the brood. In those cases, the male was identified by his unique colour-ring combination and/or captured (see [30] for further details).

Birds were individually marked with a numbered metal ring (males and females) and a unique combination of colour rings (only males), measured for morphological traits and aged. The exact age was known for many locally-born birds (ca. 53%) [47]. Unringed birds first caught as breeding adults were aged as first-year or older based on their plumage [47, 48]. Birds were weighed (± 0.1 g) and measured for tarsus length (± 0.5 mm), wing length (± 0.5 mm) and forehead patch surface (±0.01 mm^2^). The area of the forehead patch (in both males and females) was calculated as patch height × width. In males, dorsal mantle colour ranges from black to pale-brown. This trait was visually estimated as the percentage of black feathers (in relation to brown, grey or white feathers) in the area between the wings, excluding the rump [49, 50]

All fledglings were ringed when 13 days-old, which allowed us to establish their fate (whether they recruited or not) in the following years and assess for each female their number of recruits and the lifetime reproductive success of the offspring (i.e. grand-offspring). The studied pied flycatcher population shows strong natal and breeding site fidelity, with up to 22% of the fledglings returning to the natal site, this being among highest rates reported for the species [47, 51]. Further, there is no familial resemblance in dispersal patterns, thus offspring of individuals with high propensity to stay/disperse are not more prone to stay/disperse [52]. Finally, breeding outside the study plots, either in the surroundings (as indicated by surveys conducted during the breeding seasons) or in more distant areas, including other study populations of Iberian flycatchers (as indicated by ring recoveries), is an extremely rare event (pers. obs.; [53]). This suggests that most surviving fledglings reproduce in the study population. It is therefore reasonable to assume that the recruits in the population are an unbiased sample of all recruits.

### Determination of breeding status

This study covers social behaviours, for patterns of extra-pair paternity in the study population see e.g. [29, 45]. A male was classified as socially polygynous when it had been repeatedly identified and/or captured in another nest while feeding nestlings. All polygynous males were bigynous. During each breeding season, females were assigned to one of the following four groups according to their social mating status: (1) Females of socially monogamous males (n = 2358 nests); (2) primary females of socially polygynous males (n = 105); (3) secondary females of socially polygynous males (n = 105), defined as those with a later laying date than the primary female and with any degree of male assistance in chick feeding; (4) females without male assistance (n = 59), when no male was ever observed assisting with nestling feeding across monitoring sessions. Unassisted females may be secondary females, but their mates may also have abandoned or become widowed after pairing. Since female mating status and male identity in unassisted nests is unclear, data from these nests/individuals were excluded from analyses examining the phenotypic determinant of social polygyny. Note, however, that the pairing status is known for the vast majority of females (98% on average during the study period), and a two-year genetic study in this population suggests that more than half of nests without male assistance are not sired by polygynous males [30, 36]. Therefore, the exclusion of unassisted females is unlikely to induce severe biases in the results of this study.

Secondary females of polygynous males by definition reproduce later than their primary female. However, our categorical classification of females allowed us to tease apart the association between breeding dates and the probability of becoming secondary since (i) our approach is based on a local scale and the neighbours of the primary and secondary females of a polygynous male are different by definition (see below). (ii) There is a wide variation in phenology (ca. 2 months) within breeding seasons and in the intervals between the breeding dates of polygynous male’s females (1–23 days; [30]).

#### Factors influencing the probability of social polygyny

Taking advantage of our individual monitoring over time within and among years, we were able to construct local networks of breeding pairs to investigate why particular individuals engage in polygynous matings with each other. We used a spatially explicit method proposed by [26] (see also [30]), in which all breeding individuals in the population (except the social pair or primary female in the case of polygynous males) are initially considered as potential mates for each focal individual. Thus, all possible male-female combinations that may tentatively occur in a population each year are generated (i.e. male1–female2, male1–female3, etc.; male2–female1, male2–female3, etc.). For every male-female combination generated, we accounted for specific attributes of the pair (e.g. distance between their nests) and the individuals (e.g. their phenotypic traits) potentially influencing the probability of polygyny for that given combination, i.e. the probability that the male becomes polygynous with that particular female and that of the female of becoming secondary with that particular male. This information was then analysed using a Generalized Linear Mixed Model (GLMM) with binomial distribution (no/yes—coded for the combination that includes the actual polygynous male and the secondary female identified in the field).

To examine the influence of individual quality on the probability of polygyny in males and females, we focused on phenotypic traits (age, tarsus and wing lengths as well as males’ dorsal colour and males’ forehead patch size; see Table 1) that may influence success in mating and intra-sexual contexts in flycatchers (e.g. [34, 35, 45, 54, 55]). In addition, we simultaneously controlled for other potentially important socio-ecological parameters (e.g. breeding distance; [30]) that may affect the probability of polygyny.

**Fig. 1:**
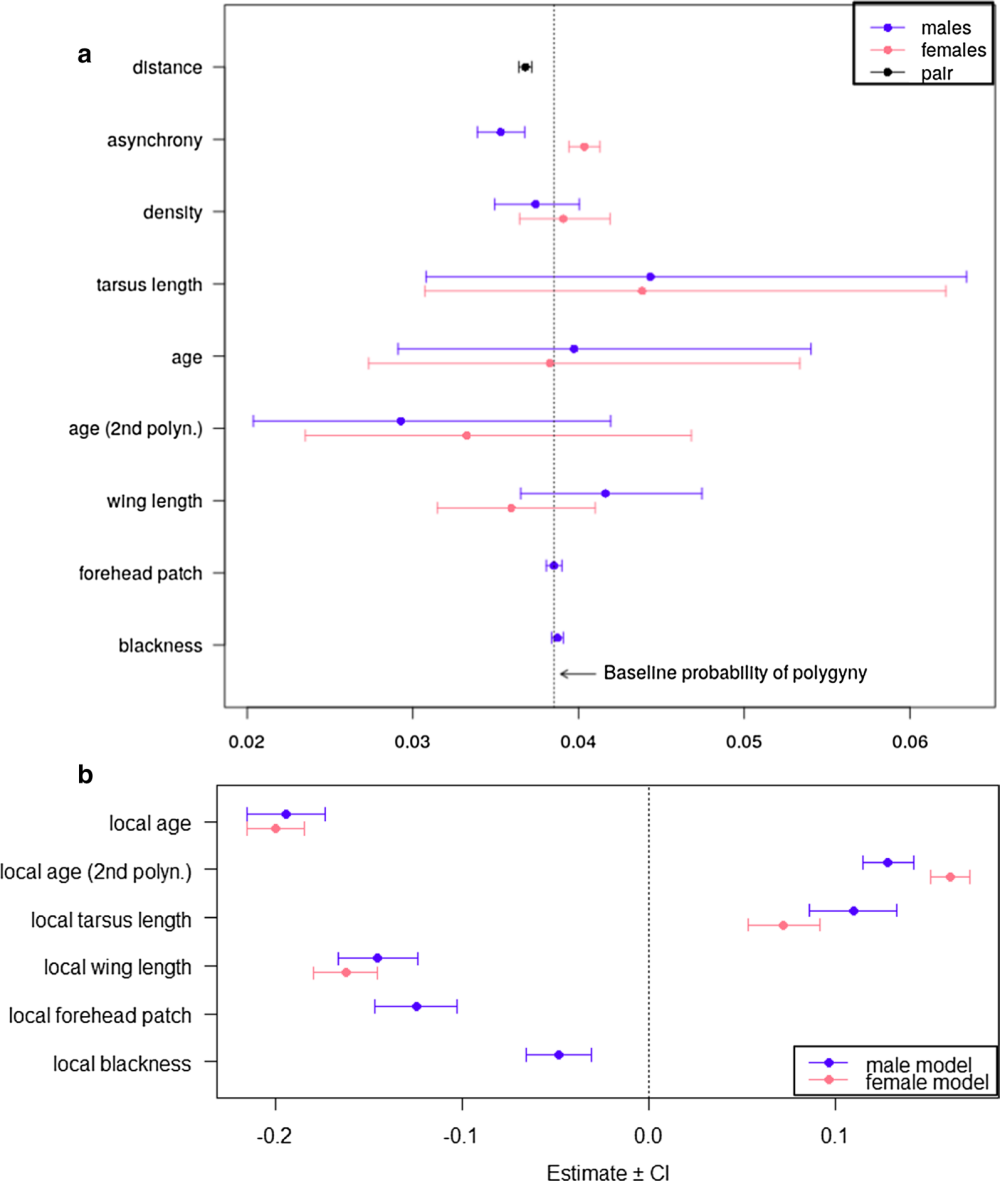
**a** Effects of the phenotypic and local breeding contexts on the probability of social polygyny of males and females. **b** Effect of the phenotype on the local breeding asynchrony of males and females, measured as the mean difference (days) in the onset of laying relative to the neighbours. Positive values indicate that individuals bred later relative to the neighbours. Phenotypic traits in males and females are compared in relation to their neighbours. Note that the forehead patch size and the plumage blackness apply only to males. The effect of age was estimated by creating two orthogonal vectors for the first and second polynomial from the original variable (with function "poly" in the R software), and thus the resulting estimates cannot be immediately interpreted biologically (see main text for details).

We defined breeding distance as the Euclidean distance (m) between the focal male and female and used Thiessen polygons to define neighbourhoods. Thiessen polygons divide the breeding area into discrete regions (the territories) within each breeding season based on the distance between occupied nest-boxes. Based on their relative position, individuals were thus classified as 1st order neighbours (direct), 2nd order neighbours, and so on (for further details see: [26, 56]). Under this spatially-explicit model, the term neighbourhood refers to all the individuals breeding at distances, in number of territories, closer or equal to the distance between the focal male-female combination. Therefore, if any specific male-female combination was constituted by individuals breeding at a distance of three territories, the variables at the neighbourhood level (e.g. breeding date) were compared relative to all the 1st, 2nd and 3rd order neighbours of the male (male neighbourhood) or the female (female neighbourhood). The use of distinct criteria to define neighbourhood boundaries (e.g. considering all individuals breeding at the same distance, but not closer, than the distance between the focal male-female combination as neighbours; see [26]) did not qualitatively change the conclusions relative to the less restrictive approach described above (results not shown). In our case, the data set used in all analyses included only the 1st and 2nd order neighbours, because in the study population polygyny rarely (3 out of 105 cases) occurs farther than the two closest territories [30]. Therefore, the addition of pairs beyond this spatial threshold is not meaningful and may also artificially decrease the intercept (“baseline probability”; Fig. 1).

Once the spatio-temporal boundaries of each individual’s neighbourhood were established, we compared the traits of each focal individual within each male-female combination relative to neighbours (as described in [26]). Thus, for each male-female combination, we compared the traits of the male and the female to the male’s neighbours (male trait-mean (trait of all neighbouring males)) and to the female’s neighbours (female trait - mean (trait of all neighbouring females)), respectively. Likewise, we also estimated analogous variables of breeding density and synchrony at the local level. For each focal female-male combination, the local male and female breeding density was defined as the number of neighbours either of the male or the female, respectively. Local asynchrony was defined as the mean difference (days) in egg-laying (first egg) dates between the focal individual and all alternative potential pairs in the neighbourhood. Local asynchrony was estimated from the male’s perspective (relative to all other pairs surrounding the given male) and from the female’s perspective (relative to all other pairs surrounding the given female). This variable thus reflects whether a given individual breeds early or late relative to its neighbours.

#### Fitness consequences of social polygyny

We tested whether direct or indirect fitness estimates of secondary females were equal to or higher than those of monogamous females. Direct fitness of females was measured as the number of recruits produced in a given breeding season, while indirect benefits were measured as the lifetime number of fledglings produced by the offspring. Offspring lifetime reproductive success was computed for all cohorts until 2011 (included), so that there was sufficient time for all individuals to be monitored during their lifetime.

Extended fitness analyses using (i) a less conservative criterion for female classification (considering all females lacking male assistance as secondary females, as described by [32]) and (ii) the number of fledged offspring as an additional proxy of direct fitness are included in the Additional file 1. Simulations to determine the statistical power of detecting significant fitness differences between females of different mating status are also included in the Additional file 2.

### Statistical analyses

All predictors used in the models were centered and standardized (as indicated in [26]). We systematically performed several model diagnostics statistics (e.g. distribution of residuals, influential data points) to avoid misleading results based on statistical artefacts. We explored multicollinearity between potentially correlated phenotypic traits by computing Variation Inflation Factors (VIFs). These analyses did not show apparent deviations from the assumptions of linear models (e.g. all VIF values < 2; [57]), possibly because phenotypic traits are estimated in relation to those of the neighbours (see above and [26]).

#### Factors influencing the probability of social polygyny

To investigate the factors influencing the probability of polygyny, we used a GLMM with binomial distribution and logit scale. We used the occurrence of polygyny (coded as “yes” or “no”) for each male-female combination (See “Methods”) as response variable. We included 15 explanatory terms as detailed below. First, five variables defining the breeding contexts of individuals for both males and females: breeding distance (defined for the nests’ location of the male-female combination), the male’s local asynchrony (based on the laying onset of the (primary) female) and his number of neighbours, as well as the female’s local asynchrony and her number of neighbours. Second, eight morphological variables for males (4) and females (4) relative to their neighbours: tarsus length, wing length, age, and squared age. We consider squared age because young and old individuals could have lower success in polygyny, for example, due to experience- or senescence-related effects. Finally, we included the expression of 2 secondary male sexual traits relative to their neighbours: dorsal plumage colour, size of the white forehead patch. Male and female body masses were not considered in the analyses, because this trait fluctuates considerably throughout the season [58] and most individuals were captured while feeding their nestlings, after the sensitive period determining success in social polygyny. Male and female identities were included as random intercepts in the models to account for individuals appearing multiple times in the dataset [26]. Year was not included as a random term because of model fitting issues.

The output of the full model above (Table 2) showed that male and female asynchronies relative to the neighbours were the best predictors of the probability of polygyny (besides breeding distance, included in the model to control for spatial autocorrelation and discussed elsewhere; [30]). Thus, we fitted two additional GLMMs, one per sex, to investigate whether the phenotype of individuals (as a proxy of their quality) predicted their breeding date relative to neighbours (as the response variable) and thus mediated the observed association between local breeding phenology and probability of polygyny. In these two models, we considered the same phenotypic traits described above for each sex, along with the quadratic term of age to control for an aging effect in the timing of breeding [59]. Year and individual identity were considered random terms.

Opposing effects of intra- and between individual processes could mask the effect of morphological traits on the probability of polygyny at the local level. However, a preliminary analysis of this possibility using the within-subject centering approach [60] suggested otherwise (data not shown). Concerning the models on local asynchronies, given that individuals appearing (i.e. breeding) only once prevail in our dataset (ca. 60%), and that inter-annual changes in phenotypic traits are minor compared to life history or behavioural traits, is reasonable to assume that the detected relationships mostly reflect between-individual effects. Note that a detailed examination and discussion of the within- and between-individual effects goes beyond the objectives of this study.

### Fitness consequences of social polygyny

We used separate GLMMs to compare the direct (number of recruits) and indirect fitness benefits (sum of lifetime number of fledglings raised by the offspring) of females in relation to their mating status (class variable: monogamous, primary and secondary). Breeding date was included in the models as a control variable. We used the corrected Akaike information criterion (AICc) to sequentially compare the fit of different error distributions and different random structures (Additional file 3). For the analyses of the number of recruits, the most supported model had a Conway-Maxwell Poisson distribution, year as a random intercept and zero inflation (Additional file 3). For the analyses of the lifetime number of fledgings the most supported model had a negative binomial distribution, year as a random intercept and zero inflation (Additional file 3). Our results are based on these two models.

Statistical analyses were performed in R version 3.6.2 [61] with the packages *lme4* [62], *glmmTMB* [63], *bbmle* [64], *lmerTest* [65] and rptR [66]. The package DHARMa [67] and the VIF function of the package *car* (Fox and Weisberg [68]) were used for model diagnostics. Thiessen polygons were calculated with the packages *expp* [69] and *spatstat* [70], whereas phenotypic traits relative to the neighbours were compared using the package *expp*. To model the second polynomial of age statistically correct, we use the function "poly" (stats package [61]), which creates two orthogonal vectors for the first and second polynomial from the original variable. Note that the resulting estimates cannot be immediately interpreted biologically.

## Results

In both males and females, the probability of polygynous mating was related to the degree of breeding asynchrony, but not to their phenotype relative to the neighbours (Fig. 1a and Table 2). Specifically, early-breeding males and late-breeding females in the neighbourhood increased their chances of becoming polygynous or being secondary, respectively.

**Table 2:** Effect of phenotypic traits and local breeding contexts on the probability of polygyny (0/1) for a given male-female combination. Phenotypic traits in males and females are compared in relation to their neighbours to investigate mating patterns at the neighbourhood level. The effect of age was estimated by creating two orthogonal vectors for the first and second polynomial from the original variable (with function "poly" in the R software), and thus the resulting estimates cannot be immediately interpreted biologically (see main text for details)

Random effects	Variance	Std. Dev
Female ID	< 0.001	< 0.001
Male ID	< 0.001	< 0.001

Fixed effects:	Estimate	Std. error	z	P
Intercept	− 3.22	0.26	− 12.15	< 0.001
Distance	− 0.05	0.01	− 8.50	< 0.001
Female's breeding date	0.05	0.01	3.91	< 0.001
Female's number of neighbours	0.01	0.04	0.41	0.69
Female's tarsus length	0.13	0.19	0.72	0.47
Female's age	− 0.01	0.18	− 0.04	0.97
Female's age (quadratic)	− 0.15	0.18	− 0.84	0.40
Female's wing length	− 0.07	0.07	− 1.03	0.30
Male's breeding date	− 0.09	0.02	− 4.23	< 0.001
Male's number of neighbours	− 0.03	0.04	− 0.85	0.40
Male's tarsus length	0.15	0.19	0.76	0.45
Male's age	0.03	0.16	0.20	0.85
Male's age (quadratic)	− 0.28	0.19	− 1.50	0.14
Male's forehead patch size	0.00	0.01	0.00	1.00
Male's black plumage (dorsal)	− 0.01	0.00	1.13	0.26
Male's wing size	0.08	0.07	1.16	0.25

To further examine the role of the phenotype, we investigated in both sexes whether the breeding date at the neighbourhood level was predicted by the phenotype relative to that of the neighbours. We found in males that darker individuals, with larger forehead patches and wings but short tarsi, were more likely to breed earlier than their neighbours (Fig. 1b and Table 3). The breeding phenology of males within the neighbourhood was also related to the quadratic age, suggesting that youngest and oldest males breed later than their middle-aged neighbours (2–4 years; Fig. 1b and Table 3). For females, we found that those with large wings and in middle-age classes were more likely to breed locally earlier (Fig. 1b and Table 3).

**Table 3:** Effect of phenotypic traits on the local breeding asynchrony of males (a) and females (b) measured as the mean difference (days) in the onset of laying relative to the neighbours. Positive values indicate that individuals bred later relative to the neighbours. Phenotypic traits in males and females were also compared in relation to their neighbours. The effect of age was estimated by creating two orthogonal vectors for the first and second polynomial from the original variable (with function "poly" in the R software), and thus the resulting estimates cannot be immediately interpreted biologically (see main text for details)

(a) Males		
Random effects	Variance	Std. dev
Male ID	34.10	5.84
Year	1.51	1.23
Residual	12.6	3.55

Fixed effects:	Estimate	Std. error	df	t value	P
Intercept	− 1.20	0.35	28.17	−3.39	< 0.001
Male's tarsus length	1.18	0.13	14160	9.13	< 0.001
Male's age	− 1.59	0.09	1715	−18.22	< 0.001
Male's age (quadratic)	1.04	0.06	16520	18.37	< 0.001
Male's forehead patch size	− 0.05	0.00	15110	−10.98	< 0.001
Male's black plumage (dorsal)	− 0.01	0.00	16540	5.53	< 0.001
Male's wing size	− 0.54	0.04	15730	−13.34	< 0.001

(b) Females		
Random effects	Variance	Std. Dev
Female ID	44.85	6.70
Year	1.37	1.17
Residual	13.47	3.67

Fixed effects:	Estimate	Std. error	df	t value	P
Intercept	0.01	0.34	30.33	0.02	< 0.001
Female's tarsus length	0.90	0.12	19090	7.38	< 0.001
Female's age	− 2.08	0.08	1922	− 25.03	< 0.001
Female's age (quadratic)	1.68	0.05	20190	30.84	< 0.001
Female's wing size	− 0.71	0.04	20400	− 18.76	< 0.001

**Fig. 2:**
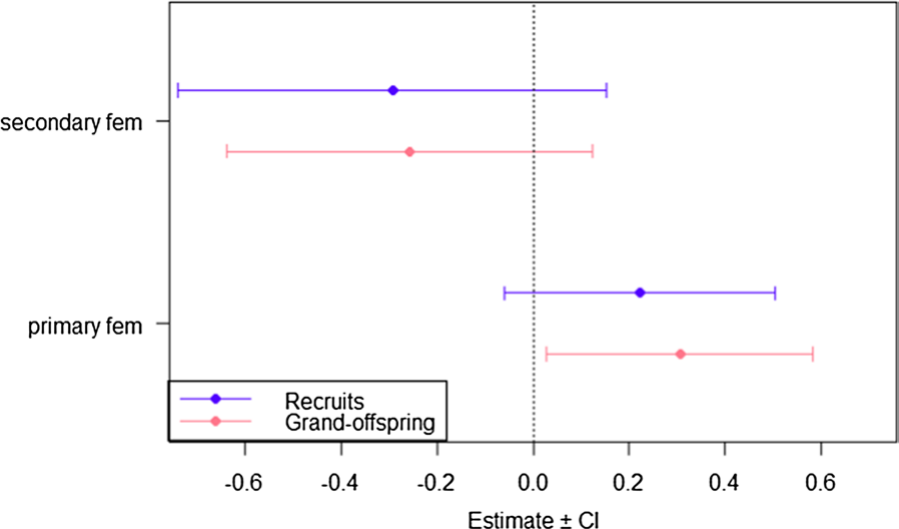
Direct (number of recruits) and indirect (number of grand-offspring) fitness estimates for monogamous, primary and secondary females. Estimated coefficients of primary and secondary females are interpretable as differences in the response variable relative to monogamous females, which was set as the reference level.

Both direct (no. of recruits) and indirect (no. of grand-offspring) proxies of fitness for secondary females were similar to monogamous females (recruits _[sec vs. mon]_: β = − 0.29, SE = 0.23, p = 0.20; grand-offspring _[sec vs. mon]_: β = −0.26, SE = 0.19, p = 0.19; Fig. 2). By contrast, primary females had similar direct fitness, but higher indirect benefits than monogamous females (recruits _[prim vs. mon]_: β = 0.22, SE = 0.14, p = 0.12; grand-offspring _[prim vs. mon]_: β = 0.30, SE = 0.14, p = 0.03; Fig. 2). Breeding date had a significant negative effect on direct fitness, but not on indirect fitness (respectively, β = − 0.29, SE = 0.05, p < 0.01 and β = − 0.05, SE = 0.05, p = 0.23).

## Discussion

Using a spatially explicit approach based on local networks of breeding pairs, we show that breeding date within a neighbourhood mediated the probability of polygyny in male and female pied flycatchers. Middle-aged individuals (2–3 years), with larger wings and, in the case of males, with more elaborate sexual ornaments compared to neighbours, started breeding earlier in the neighbourhood. Secondary and monogamous females experienced similar direct and indirect fitness, but the evidence that mating polygamously is adaptive for females is inconclusive as these findings are open to alternative explanations.

We have previously shown that early breeding males at the population level are more likely to become polygynous in our study system [30]. This study indicates that this pattern stands at the neighbourhood level, even after accounting for phenotypic traits that are relevant in intra and inter-sexual contexts. It is possible that an early reproductive phenology, implying an early arrival, relative to the neighbours increased the male’s chances of acquiring multiple nearby nest-boxes, of being more frequently visited by prospecting females and therefore, of eventually becoming polygynous. In contrast, late females within the neighbourhood might be time-constrained in selecting a mate and would mostly find already mated neighbours, thus increasing their probability of becoming secondary.

Considering that the probability of being involved in social polygyny in male and female pied flycatchers is determined by their local breeding time, we investigated whether variation in individual phenology relative to neighbours was mediated by the phenotype. The rationale is that reproductive phenology provides reliable information about an individual’s quality, since individuals in prime condition are expected to arrive and breed earlier than low-quality individuals (e.g. [71–74]). Several traits indicative of individual quality had an independent effect on the reproductive phenology of males and females (discussed below), which suggests that multiple cues, possibly signalling different aspects of individual quality (reviewed in [75]), affect male-male contests, mating decisions and, thereby, the probability of polygyny in pied flycatchers. Despite the relationships of breeding dates with both polygyny and phenotype, we found no direct association between phenotype and polygyny. This may be due to noise around each of the estimated variables, leading by default to small effects sizes and low statistical power (i.e. a relationship present, but not detected). Alternatively, the part of the variation in breeding dates that drives the relationship between breeding date and phenotype may be different from the variation in breeding dates that drives the relationship between breeding date and polygyny (relationship no present).

The breeding date relative to neighbours in males showed a bell-shaped variation with age, which resembles the population-wide pattern of breeding date reported in other bird species [59, 71, 73, 76]. It is possible that delaying breeding allowed young and senescent individuals to reduce aggressions by high-performing and still-unpaired neighbouring males [77]. Differential local phenology by age might also be related to carry-over effects from the non-breeding season, such as the occupation of poor-quality wintering territories or the poorer knowledge of the migratory routes in younger individuals [76, 78]. Regardless of the mechanisms underlying this reproductive pattern, our findings suggest that middle-aged males (2–3 years old) would be able to breed locally early and acquire secondary females to maximize their fitness [79]. Similarly, middle-aged females minimized the probability of becoming secondary [36, 39, 80], even though early breeding might not preclude the possibility of sharing a mate by increasing the probability of becoming primary [38].

Local phenology in both sexes was also influenced by the own phenotype. In particular, the probability of breeding locally early was higher in males with relatively larger wings, larger forehead patches, blacker plumages and shorter tarsi than their neighbours. Large wings probably improve the flight performance of migrants, favouring a relatively early arrival and breeding [35, 81, 82]. Tarsus length, however, is positively related to within-sex dominance in territorial disputes [37] and therefore, a negative relationship between tarsus length and breeding time might be expected *a priori*. Nonetheless, the benefits of having large wings, by enabling birds to arrive at the breeding areas when the number of competitors is still limited, may have overridden any potential disadvantage of having a short tarsus during competition over nest sites (note that the correlation matrix of the model indicates that both traits are slightly, but negatively correlated r = − 0.14). As in males, locally, early-breeding females also had larger wings and smaller tarsi than late breeders. Dorsal coloration is a sexually selected and reliable signal of individual quality in male pied flycatchers as darker birds have higher pairing success [35, 55], larger song repertoires [83] and feed their nestlings more frequently [84]. Further, darker plumages are positively associated with aggressiveness, territoriality and exploratory behaviour [49, 85]. Thus, dark males might have a competitive advantage over nest sites and mates. Finally, the white forehead patch functions as a sexual and social signal in pied flycatchers (e.g. [34, 45]). Males with large forehead patches enjoy competitive [34, 86] and mating advantages via EPP [45], so males displaying enhanced sexual traits are expected to have a competitive advantage both within inter- and intra-sexual competition contexts.

### Implications on the main adaptive hypothesis of social polygyny

The relationship between phenotypic quality, local breeding phenology and, subsequently, success in polygyny raises the question of whether females benefit from social polygyny in terms of fitness. We did not find significant differences between monogamous and secondary females in any of the direct or indirect fitness proxies considered. Such results might reflect a compensatory mechanism in agreement with the two main adaptive hypotheses on social polygyny: the polygyny threshold model (PTM; [3, 17]) and the sexy son hypothesis (SSH; [14]). In their original formulation, the critical predictions of these two models are that the number of offspring (PTM) and grand-offspring (SSH) is similar or higher for females mated with a polygynous male than for those mated with a monogamous male. However, inferential limitations imposed by the relatively small sample size of secondary females, even when considering long temporal series (Additional file 2), claim for some caution before categorically accepting/rejecting any of these two hypotheses. In addition, results may vary depending on the criteria to categorize female mating status. In fact, when we repeated the analyses with another classification criterion and considered, as in previous work (e.g. [32]), all females without male assistance as secondary, secondary females had significantly lower indirect fitness than monogamous females (Additional file 1). However, it is important to note that, in the study population, there is evidence indicating that a large fraction of females without assistance is not polygamously-mated (see “Methods”), hence considering them as such may lead to misleading interpretations. Finally, it is possible that the PTM and SSH do not reflect average differences in fitness between females of different mating status at the population level, but rather operate at the individual level, as a female’s preference and benefits might depend on the quality of the specific males currently available to her [31]. It should also be noted that EPP may contribute to increase the indirect fitness of secondary females, thus levelling fitness between female categories. Although further data are needed to evaluate adequately this possibility, this is a rather unlikely scenario, as EPP levels are relatively low in the study population (as indicated by a two-year study [29]) and secondary females should systematically incur in EPP and there is no evidence of the latter in the study population [45]. Regardless of the underlying reasons, we have not found convincing support for an adaptive explanation of social polygyny under the umbrella of the PTM and SSH hypotheses which is in line with previous works (reviewed in [31, 87]), including two empirical studies in the pied flycatcher [32] and the closely related collared flycatcher, *F. albicollis* [12].

## Conclusions

Using a comprehensive analytical framework, we have shown phenology-mediated effects of the phenotype on the probability of becoming polygynous and secondary in pied flycatchers. However, as in previous studies, we found no convincing support for the adaptive value of mating polygamously in females. Leaving aside the possible inferential limitations imposed by the (relatively) low frequency of secondary females, an interesting possibility to explain the low empirical evidence for the adaptive hypothesis on social polygamy, namely PTM and SSH, could be due to the wrong focus on population instead of individual benefits. Thus, further work investigating whether the fitness consequences of social polygyny (PTM, SSH) vary at the individual level (rather than at the population level) in combination with experimental studies (e.g. manipulating the cost/benefits due to mate sharing by simultaneously dealing with male quality and mating status; Slagsvold and Drevon [88]) are required to elucidate whether social polygyny in this and other species arises as a result of sexual conflict.

## Supplementary Information

**Additional file 1.** Complementary fitness analyses.

**Additional file 2.** Simulation analyses.

**Additional file 3.** Model selection.

## Data Availability

The datasets used and/or analysed during the current study are available from the corresponding author on reasonable request.
