# Supplementary material for: Phenology-mediated effects of phenotype on the probability of social polygyny and its fitness consequences in a migratory passerine
Source: BMC Ecol Evol. 2021 Apr 13;21:55. doi: 10.1186/s12862-021-01786-w (PMC8042933; doi:10.1186/s12862-021-01786-w)
Supplement: Supplementary file 1 — Additional file 1. Complementary fitness analyses. [file 12862_2021_1786_MOESM1_ESM.docx]

**Additional file 1**

**Complementary fitness analyses**

Previous works on social polygyny have used different proxies of fitness and/or followed different criteria to classify females according to their mating status (e.g. by including/excluding females without male assistance as secondary females; [12, 31, 32, 89]. These discrepancies might affect the conclusions about the fitness consequences of social polygyny for females [31, 89]. In this study we used a conservative approach by only considering confirmed cases of social polygyny (i.e. secondary females with males assistance) as, based on a two-years genetic study, we are aware that a fraction of nests without male assistance are not sired by polygynous males (see methods). For completeness, we performed variants of these fitness analyses i) using the number of fledged offspring as an additional proxy of direct fitness and ii) considering all females lacking male assistance as secondary females (as described by Huk and Winkel 2006).

Models structure using number of fledging and selection of final model resembled those described in the main text. The number of fledglings did not differ between secondary and monogamous females (fledges _[sec vs. mon]_: β = - 0.03, SE = 0.04, p = 0.43; fledges _[prim vs. mon]_: β = - 0.03, SE = 0.03, p = 0.29), whereas breeding date had a negative effect on direct fitness (β = - 0.08, SE = 0.01, p < 0.01).

We also estimated the direct (number of recruits and of fledges) and indirect (number of grand-offspring) benefits for females of social polygyny following the classification made by Huk and Winkel 2006. This classification considers two main groups of females according to their mating status: monogamous females and secondary females, the latter including confirmed secondary females as well as all females without male assistance (we are aware that this classification assigns incorrectly the true mating status of a fraction of individuals in our study population; see methods). Results using this classification were similar to those excluding females without male assistance from the group of secondary females (see main text), with the only difference that the lower direct fitness of secondary females over that of monogamous females becomes significant (recruits_[sec vs. mon]_: β = - 0.49, SE = 0.19, p = 0.01; recruits_[prim vs. mon]_: β = 0.23, SE = 0.15, p = 0.12; fledges_[sec vs. mon]_: β = - 0.16, SE = 0.03, p < 0.01; fledges_[prim vs. mon]_: β = -0.02, SE = 0.03, p = 0.47; grand-offspring_[sec vs. mon]_: β = - 0.16, SE = 0.16, p = 0.33; grand-offspring_[prim vs. mon]_: β = 0.31, SE = 0.14, p = 0.03).
