# Supplementary material for: Phenology-mediated effects of phenotype on the probability of social polygyny and its fitness consequences in a migratory passerine
Source: BMC Ecol Evol. 2021 Apr 13;21:55. doi: 10.1186/s12862-021-01786-w (PMC8042933; doi:10.1186/s12862-021-01786-w)
Supplement: Supplementary file 2 — Additional file 2. Simulation analyses. [file 12862_2021_1786_MOESM2_ESM.docx]

**Additional file 2**

Simulation-based statistical power of detecting different scenarios of effect sizes (β) meant as the difference in recruits between monogamous vs primary females and monogamous versus secondary females. The model used to simulate data under different effect sizes is the most supported model in the analysis of the effect of mating status on the number of recruits (see main text), that is a GLMM with a Conway-Maxwell-Poisson distribution (family link = log) and zero inflation. The effect sizes are expressed in the link scale. All the parameters estimates have been maintained equal to those obtained from the most supported model except the values of the effect sizes of *Mon vs Prim* and *Mon vs Sec* that have been set to the values reported in the table. The first ten scenarios (sc1 – sc10) are for the data that do not contain unassisted females. The 11^th^ scenario is for the unassisted females considered secondary females (see main text and SF1). In the 10^th^ and 11^th^ scenarios, we have left all the parameters estimates as those obtained from the most supported model. Note that the relatively high statistical power of β_Mon vs Sec_ in sc11 is due to the larger effect size, but also to the larger sample size (additional 53 females classified as “secondary”).

| **Scenarios** | **β_Mon vs Prim_** | **β_Mon vs Sec_** | **power (%) β_Mon vs Prim_** | **power (%) β_Mon vs Sec_** |
| --- | --- | --- | --- | --- |
| **sc1** | 0.1 | -0.1 | 31 | 15 |
| **sc2** | 0.2 | -0.1 | 35 | 18 |
| **sc3** | 0.5 | -0.1 | 38 | 17 |
| **sc4** | 0.1 | -0.2 | 33 | 19 |
| **sc5** | 0.2 | -0.2 | 37 | 14 |
| **sc6** | 0.5 | -0.2 | 38 | 20 |
| **sc7** | 0.1 | -0.5 | 37 | 21 |
| **sc8** | 0.2 | -0.5 | 32 | 19 |
| **sc9** | 0.5 | -0.5 | 41 | 24 |
| **sc10** | 0.22 | -0.29 | 36 | 16 |
| **sc11** | 0.22 | -0.49 | 36 | 73 |
