# Supplementary material for: Phenology-mediated effects of phenotype on the probability of social polygyny and its fitness consequences in a migratory passerine
Source: BMC Ecol Evol. 2021 Apr 13;21:55. doi: 10.1186/s12862-021-01786-w (PMC8042933; doi:10.1186/s12862-021-01786-w)
Supplement: Supplementary file 3 — Additional file 3. Model selection. [file 12862_2021_1786_MOESM3_ESM.docx]

Additional file 3

During analyses of fitness, we used the corrected Akaike information criterion (AICc) to sequentially compare the fit of (i) different error distributions (negative binomial, Poisson, Conway-Maxwell Poisson), (ii) different random structures (initially three random intercepts: year, mother identity, and social father identity) and (iii) zero-inflation [90].

For the analysis of direct fitness (number of recruits), the most supported model had a Conway-Maxwell Poisson distribution, year as a random intercept and zero inflation. For the analysis of indirect fitness benefits (number of grand-offspring), we found most AICc-based support for a model with a negative binomial distribution, year as a random intercept and zero inflation. Results shown in the main text are based on these two models.

|  | **Direct Fitness** | |  |  | **Indirect Fitness** | |
| --- | --- | --- | --- | --- | --- | --- |
|  | dAICc | df |  |  | dAICc | df |
| **Distribution** |  |  |  | **Distribution** |  |  |
| Conway-Maxwell Poisson | **0** | **9** |  | Neg. Binomial | **0** | **9** |
| Poisson | 5.3 | 8 |  | Poisson | 75.3 | 8 |
| Neg. Binomial | NA | 9 |  | Conway-Maxwell Poisson | 18.5 | 9 |
| **Zero inflation** |  |  |  | **Zero inflation** |  |  |
| Neg. binomial + zero inflation | **0** | **9** |  | Neg. binomial + zero inflation | **0** | **9** |
| Neg. binomial | 7.7 | 8 |  | Neg. binomial | 333.1 | 8 |
| **Random Structures** |  |  |  | **Random Structures** |  |  |
| Year | **0** | **7** |  | Year | **0** | **7** |
| Year + Female identity | 1.6 | 8 |  | Year + Male identity | 0.9 | 8 |
| Year + Male identity | 2 | 8 |  | Year + Female identity | 2 | 8 |
| Year + Male + Female identities | 3.6 | 9 |  | Year + Male + Female identities | 3 | 9 |
| No random factors | 22.8 | 6 |  | No random factors | 4.4 | 6 |
| Female identity | 24.3 | 7 |  | Female identity | 5.8 | 7 |
| Male + Female identities | 26.4 | 8 |  | Male + Female identities | 6 | 8 |
